# Relationship between early language skills and the development of inattention/hyperactivity symptoms during the preschool period: Results of the EDEN mother-child cohort

**DOI:** 10.1186/s12888-016-1091-3

**Published:** 2016-11-08

**Authors:** Hugo Peyre, Cedric Galera, Judith van der Waerden, Nicolas Hoertel, Jonathan Y. Bernard, Maria Melchior, Franck Ramus, on behalf of the EDEN Mother-Child Cohort Study Group, I. Annesi-Maesano, J. Y. Bernard, J. Botton, M. A. Charles, P. Dargent-Molina, B. de Lauzon-Guillain, P. Ducimetière, M. de Agostini, B. Foliguet, A. Forhan, X. Fritel, A. Germa, V. Goua, R. Hankard, B. Heude, M. Kaminski, B. Larroque†, N. Lelong, J. Lepeule, G. Magnin, L. Marchand, C. Nabet, F. Pierre, R. Slama, M. J. Saurel-Cubizolles, M. Schweitzer, O. Thiebaugeorges

**Affiliations:** 1 Laboratoire de Sciences Cognitives et Psycholinguistique (ENS, EHESS, CNRS), Ecole Normale Supérieure, PSL Research University, 29 rue d’Ulm, 75005 Paris, France; 2 Department of Child and Adolescent Psychiatry, Robert Debré Hospital, APHP, Paris, France; 3 Department of Child and Adolescent Psychiatry, Charles Perrens Hospital, Bordeaux, France; 4 The Bordeaux School of Public Health (Institut de Santé Publique, d’Epidémiologie et de Développement), Centre INSERM U897, Epidemiology-Biostatistics, Bordeaux, France; 5 INSERM, Institut Pierre Louis d’épidémiologie et de Santé Publique (IPLESP UMRS 1136), Department of Social Epidemiology, Sorbonne Universités, UPMC Univ Paris 06, Paris, 75012 France; 6 Department of Psychiatry, Corentin Celton Hospital, APHP, Issy-les-Moulineaux, France; 7 Paris Descartes University, PRES Sorbonne Paris Cité, Paris, France; 8 INSERM UMR 894, Psychiatry and Neurosciences Center, Paris Descartes University, PRES Sorbonne Paris Cité, Paris, France; 9 Epidemiology and Biostatistics Sorbonne Paris Cité Center (CRESS), Developmental Origins of Health and Disease (ORCHAD) Team, INSERM UMR 1153, F-94807 Villejuif, France

**Keywords:** ADHD, Language, Structural equation modeling, Preschool

## Abstract

**Background:**

This study aims to examine bidirectional relationships between children’s language skills and Inattention/Hyperactivity (IH) symptoms during preschool.

**Method:**

Children (*N* = 1459) from the EDEN mother-child cohort were assessed at ages 3 and 5.5 years. Language skills were evaluated using the WPPSI-III, NEPSY and ELOLA batteries. Children’s behavior, including IH symptoms, was assessed using the parent-rated Strengths & Difficulties Questionnaire (SDQ). Using a Structural Equation Modeling (SEM) approach, we examined the relationship between language skills and IH symptoms, as well as potential mediating processes.

**Results:**

SEM analyses indicated a small negative effect of language skills at 3 years on ADHD symptoms at 5.5 years after adjusting for IH symptoms at 3 years (β =−0.12, SE = 0.04, *p*-value = 0.002). Interpersonal difficulties did not mediate the relationship between early language skills and later IH symptoms, nor was this association reduced after adjusting for a broad range of pre- and postnatal environmental factors and performance IQ. Among different language skills, receptive syntax at 3 years was most strongly related to IH symptoms at 5.5 years.

**Conclusions:**

Poor language skills at age 3 may predict IH symptoms when a child enters primary school. Implications for the understanding and the prevention of the co-occurrence of language disorders and ADHD are discussed.

**Electronic supplementary material:**

The online version of this article (doi:10.1186/s12888-016-1091-3) contains supplementary material, which is available to authorized users.

## Background

Attention Deficit Hyperactivity Disorder (ADHD) is a common childhood disorder characterized by symptoms of inattention, hyperactivity and impulsivity, that significantly impact many aspects of behavior as well as performance, both at school and at home [1, 2]. Understanding the mechanisms by which such behavioral problems can develop may have important implications on early assessment, prevention, and treatment.

Developmental difficulties rarely occur in isolation [3]. A close relationship between the development of Inattention/Hyperactivity (IH) symptoms and language skills has been consistently reported [4, 5]. Cross-sectional studies found that children with ADHD have an increased prevalence of language impairments [6, 7]. Several difficulties in linguistic skills have been reported among children with ADHD, particularly with regards to expressive language skills: phonology, vocabulary, syntax and pragmatic [8–11]. Although data on this are somewhat inconsistent [4], children with ADHD may also have deficits in receptive language skills [12]. However, in longitudinal studies the association between early IH symptoms with later language skills has been found to be weak or absent [5, 13]. Several authors have suggested that language difficulties could precede the development of ADHD and represent an early expression of the disorder [14, 15].

Conversely, cross sectional studies found that children with language impairments have an elevated prevalence of ADHD [15–18] as well as deficits in selective attention tasks, in particular in the auditory modality [19]. Longitudinal studies have reported that early language difficulties are associated with later IH symptoms during the preschool [13, 14] and school periods [5, 20, 21], even when prior levels of IH symptoms are accounted for. Recent results of longitudinal studies support a causal role of language difficulties in the development of IH symptoms [13]. Difficulties in language skills may be associated with ineffective use of self-directed speech for self-regulation, which may subsequently lead to IH symptoms (*Hypothesis 1*). Following 120 children at 30, 36, and 42 months of age, Petersen et al. [13] reported that the relationship between early language skills and later IH symptoms was mediated by language-based self-regulation during the preschool period. This result suggests that language functions (i.e., private or inner speech) may support behavioral and attentional control [22]. Nevertheless, two other hypotheses for the association between early language skills and later IH symptoms have been proposed. The link between language skills and behavioral problems may be mediated by interpersonal difficulties (*Hypothesis 2*) [23–25], as poor language skills may interfere with socialization which may then lead to IH symptoms [25]. Like all neurodevelopmental disorders, language disorders and ADHD are known to share some etiological factors (such as genetic or pre- and postnatal environmental factors) [3, 6, 26]. A last hypothesis is that the common vulnerability has a sequential expression during the development by impacting first on language skills and later on behavior (*Hypothesis 3*), creating the illusion of a directional effect between early language skills and later ADHD symptoms (i.e., heterotypic continuity [27]).

Rather surprisingly, few of the previous studies [18] have examined which aspects of early language skills are most strongly associated with the development of IH symptoms. Snowling et al. [18] reported that children’s expressive language impairment at 5.5 years was the language profile most strongly associated with ADHD in adolescence. Researchers have called for more longitudinal studies to explore the association between language difficulties and IH symptoms and specify the underlying developmental processes [28].

The preschool years are a crucial period in children’s psychological development. Previous studies support a significant instability of language skills between 3 and 5.5 years [29]. For some children, the onset of behavioral, emotional and/or social problems occurs during this period [30]. Addressing the stated research questions in preschoolers rather than in older children is of utmost importance since influences with respect to long-lasting outcomes may be more determinant during the first years of life, as suggested by the Developmental Origin of Health and Disease Hypothesis [31, 32].

In the present study, we use data from a large (*N* = 1459) prospective mother-child cohort to test bidirectional relationships between children’s language skills and inattention/hyperactivity (IH) symptoms between 3 and 5.5 years. We expect to replicate previous longitudinal studies [13, 14], which found an asymmetrical association between language skills and IH symptoms during the preschool period (i.e., the association between language skills and IH symptoms was stronger than the reverse). If the influence of early language difficulties on the development of IH symptoms is mediated by an ineffective use of self-directed speech, language tests tapping into expressive language skills should be most strongly associated with later IH symptoms (*Hypothesis 1*). Additionally, we also sought to test whether the association might be mediated by interpersonal difficulties (*Hypothesis 2*) and whether shared pre- and postnatal environmental factors might explain both language skills and IH symptoms (*Hypothesis 3*).

## Method

### Study design

We analyzed data from the EDEN prospective mother-child cohort study [33]. The primary aim of the EDEN cohort was to identify prenatal and early postnatal nutritional, environmental and social determinants of children’s health and development. Pregnant women (< twenty-fourth weeks of amenorrhea) were recruited during a prenatal visit at the Obstetrics and Gynecology department of the French University Hospitals of Nancy and Poitiers. Exclusion criteria included a history of diabetes, twin pregnancies, intention to deliver outside the university hospital or to move out of the study region within the next 3 years, and inability to speak French. The participation rate among eligible women was 53 %. Enrolment started in February 2003 in Poitiers and in September 2003 in Nancy, lasted for 27 months in each center and resulted in the inclusion of 2002 pregnant women. Compared to the National Perinatal Survey (ENP) carried out among 14,482 women who delivered in France in 2003 [34], women participating in the EDEN study had similar sociodemographic characteristics except for higher educational background (53.6 % had a high-school diploma *versus* 42.6 % in the ENP survey) and higher employment level (73.1 % were employed during pregnancy cohort *versus* 66.0 % in the ENP survey) [33, 35]. The study was approved by the Ethical Research Committee (Comité consultatif de protection des personnes dans la recherche biomédicale) of Bicêtre Hospital and by the Data Protection Authority (Commission Nationale de l’Informatique et des Libertés). Informed written consents was obtained from parents for themselves at the time of enrollment and for the newborn after delivery.

### Participants

Among the 2002 pregnant women included in the EDEN study, 1907 children were followed-up after birth, as described in detail elsewhere [33]. Some analyses of neuropsychological data collected at 2 and 3 years have previously been published [36, 37]. 1459 children completed language tests and/or the Strengths and Difficulties Questionnaire (SDQ) at ages 3 and/or 5.5 years [full sample], 914 of which had complete information on both measures [complete data sample] (the flowchart is shown in Additional file 1: Figure S1). Analyses were conducted on the full sample [*N* = 1459].

Of the 1459 children included, 52.1 % were male, mean (SD) birth weight was 3.3 (0.5) kg (4.8 % were born with a low birth weight; i.e., < 2.5 kg), and mean gestational age was 39.3 (1.7) weeks (5–6 % of births occurred preterm; i.e., < 37 weeks of gestation) (Table 1). The mean maternal age at delivery was 29.4 (SD = 4.8). The mean number of alcoholic drinks per week during pregnancy was 0.5 (SD = 1.5) and most mothers declared no alcohol consumption during pregnancy (53.8 %). 21 % of mothers regularly smoked during pregnancy. Three-quarters of participating children were breastfed for at least 3 days. In our sample, 21 % of mothers suffered from depression during pregnancy and 32 % of them up to 5 years after delivery.

**Table 1:** Summary statistics of the participating children

	Mean (SD), min-max, or %
	*N* = 1459
Male sex (vs. female)	52.1
Gestational age (weeks)	39.3 (1.7), 28–42
Birth weight (kg)	3.29 (0.50), 0.59–5.01
Maternal age at birth of child (years)	29.4 (4.8), 17–45
Tobacco consumption, %	22.8
Alcohol during pregnancy (drinks/week)	0.54 (1.46), 0–17
Breastfeeding duration (months)	3.4 (3.7), 0–13
Maternal depression during pregnancy, %	21.4
Maternal depression after birth, %	32.4
Family history of language delay, %	13.3
Household income (k€)	2.72 (1.01), 0.23–5.25
Parental education (years)	13.6 (2.3), 9–17
Number of older siblings	0.78 (0.94), 0–7
Maternal cognitive stimulation at 3 years	21.86 (4.84), 5–32
Score for family stimulation at 5.5 years	17.22 (2.29), 7–21
Recruitment center (Nancy)	47.6
Performance IQ	99.30 (13.78), 48–144
Language measures	
*At 3 years*	
Semantic fluency (ELOLA)	6.87 (3.91), 0–22
Word and nonword repetition (ELOLA)	7.63 (3.22), 0–12
Sentence repetition (NEPSY)	7.19 (3.30), 0–19
Picture naming (ELOLA)	6.96 (1.90), 0–10
Comprehension of instructions (NEPSY)	8.47 (2.98), 0–13
Age of the child at the time of testing (months)	38.05 (0.81), 34.20–42.91
*At 5.5 years*	
Non Words Repetition (NEPSY)	28.03 (8.12), 5–45
Sentence Repetition (NEPSY)	15.42 (4.10), 2–28
Information (WPPSI-3)	24.96 (2.95), 12–32
Vocabulary (WPPSI-3)	23.63 (5.65), 5–40
Word Reasoning (WPPSI-3)	16.07 (4.69), 0–27
Age of the child at the time of testing (months)	67.89 (1.85), 59.53–82.07
Strengths & Difficulties Questionnaires	
*At 3 years*	
Emotional symptoms score	6.81 (1.63), 5–14
Conduct problems score	6.20 (2.01), 3–12
Hyperactivity/inattention symptoms score	4.48 (2.25), 1–12
Peer relationship problems score	2.49 (1.50), 0–12
Prosocial behavior score	12.71 (1.68), 7–15
*At 5.5 years*	
Emotional symptoms score	7.13 (1.88), 5–15
Conduct problems score	5.37 (2.05), 2–13
Hyperactivity/inattention symptoms score	4.07 (2.42), 0–13
Peer relationship problems score	2.19 (1.33), 0–9
Prosocial behavior score	13.37 (1.68), 7–15

### Variables

#### Emotional and behavioral problems assessment

The Strengths and Difficulties Questionnaire (SDQ) [38, 39] was used to measure emotional and behavioral problems when children were aged 3 and 5.5 years. The SDQ is a 25-item scale comprising five scores covering emotional problems (items about fears, worries, misery, nervousness and somatic symptoms), conduct problems (items about tantrums, obedience, fighting, lying and stealing), IH symptoms (items on restlessness, fidgeting, the ability to concentrate, distractibility and impulsivity), peer relationships (items on popularity, victimization, isolation, friendship and the ability to relate to children as compared to adults), and pro-social behavior (items on consideration of others, the ability to share, kindness to younger children, helpfulness to other children when distressed and willingness to comfort others). Answer options for each item are: ‘Not true’ ‘Somewhat true’ or ‘Very true’, scored 0, 1 or 2, yielding a total score ranging from 0 to 10 for each subscale. Higher scores represent worse functioning except for pro-social behavior. In the present data, Cronbach’s alphas for each SDQ scale, at 3 and 5.5 years, were respectively: 0.55 and 0.60 for emotional symptoms, 0.69 and 0.73 for conduct problems, 0.70 and 0.76 for IH symptoms, 0.48 and 0.54 for peer relationship problems and 0.60 and 0.69 for prosocial behavior. These reliability estimates were similar to those found in a representative sample of 1348 French children aged 6–11 years old [39].

#### Language measures

Trained psychologists (one in each center: Nancy and Poitiers) individually assessed each child’s cognitive skills at 3 years (mean = 38.0 months; SD = 0.8) and 5.5 years (mean = 67.8 months; SD = 1.8; min = 59.5 months; max = 82.1 months) using neuropsychological tests from the WPPSI-III [40], ELOLA (Evaluation du Langage Oral de L’enfant Aphasique) [41] and NEPSY (A Developmental NEuroPSYchological Assessment) [42, 43] batteries.

At age 3, five tests were used (Table 1):

- *Semantic fluency* (ELOLA), scored as the sum of the number of animals named in 1 min plus the number of objects named in 1 min. This test is designed to measure expressive vocabulary and lexical retrieval.
- *Word and nonword repetition* (ELOLA); scored as the number of words (6 items) and nonwords (6 items) repeated correctly. This test is designed to measure phonological processing and verbal short-term memory.
- *Sentence repetition* (NEPSY) scored as the number of sentences of increasing complexity and length repeated correctly (17 items, *e.g.*, “dors bien” [“sleep well”]). This test is designed to measure verbal short-term memory and syntactic skills.
- *Picture naming* (ELOLA), scored as the number of pictures named correctly (10 items, *e.g*., “cheval” [“horse”]). This test is designed to measure expressive vocabulary.
- *Comprehension of instructions* (NEPSY), a sentence comprehension task scored as the number of correct answers by pointing at one of 8 pictures (13 items, *e.g*., “montre-moi un grand lapin” [“show me a large rabbit”]). This subtest is designed to assess the ability to receive, process, and execute oral instructions of increasing syntactic complexity.

At age 5.5, five tests were used (Table 1):

- *Nonword Repetition* (NEPSY), scored as the number of syllables repeated correctly (out of 46 syllables in 13 nonwords (*e.g*., [kiutsa], a nonword with two syllables). This test is designed to measure phonological processing and verbal short-term memory.
- *Sentence Repetition* (NEPSY), scored as the number of sentences (17 items, *e.g*., “dors bien” [“sleep well”]) repeated correctly. This test is designed to measure syntactic skills and verbal short-term memory.
- *Information* (WPPSI-III), scored as the number of correct answers (verbally or by pointing) to questions that address a broad range of general knowledge topics (34 items). This test is designed to measure language comprehension, conceptual knowledge and verbal expressive ability.
- *Vocabulary* (WPPSI-III), scored as the number of correctly defined words (25 items). This test is designed to measure receptive vocabulary, conceptual knowledge and verbal expressive ability.
- *Word Reasoning* (WPPSI-III), scored as the number of concepts correctly identified from a series of clues (28 items). This test is designed to measure language comprehension, conceptual knowledge and general reasoning ability.

Language tests were somewhat different at 3 and 5.5 years because they were selected to be age-appropriate.

The manual of WPPSI-III reports evidence of high subtest reliability (0.83 to 0.95), internal consistency, test-retest stability, and validity for all subtests [40]. The sentence repetition and comprehension of instructions tests from the NEPSY had high internal consistency (0.91 and 0.89) in a population of 3 to 4 year-old children as well as the nonword repetition and sentence repetition tests (0.80 and 0.81) in a population of 5 to 12 year-old children [43]. In the present data, Cronbach’s αs for NEPSY sentence repetition at 3 years and 5 years, comprehension of instructions at 3 years and nonword repetition at 5 years were respectively 0.72, 0.70, 0.77 and 0.80, and ELOLA battery semantic fluency, word and nonword repetition, and picture naming were respectively 0.57, 0.86 and 0.57.

#### Covariates

Analyses were adjusted for several factors potentially associated with cognitive development (Table 1). Sex, gestational age at birth, birth weight and maternal age at delivery were collected from obstetrical records; Maternal tobacco and alcohol use during pregnancy (number of drinks per week) and breastfeeding duration [36] were ascertained in maternal self-reported questionnaires. Maternal depression during pregnancy was assessed by the Center for Epidemiological Studies-Depression scale (CES-D) between 24 and 28 gestational weeks (a cut-off of 16 was used to define depression [44, 45]). We assessed postpartum depression status with the Edinburgh Postnatal Depression Scale at 4, 8 and 12 months (a cut-off of 13 was used to define depression [46, 47]) and with the CES-D at 3 and 5 years following delivery (a cut-off of 16 was used to define depression). Mothers and fathers completed questionnaires on their own history of speech and language delay. Family income (monthly household income in euros), education level (the average of the two parents’ education levels) and number of older siblings were also assessed. We included an estimate of maternal cognitive stimulation at age 3 (by averaging the weekly frequency of 8 activities; *e.g*., storytelling, singing, drawing, etc.). When children were 5.5 years old, stimulation of the child at home was assessed by the psychologist using three subscales of the Home Observation for the Measurement of the Environment Scale: language stimulation, academic stimulation, and variety of experimentations [48, 49]. Higher scores represent greater cognitive stimulation and emotional support.

At age 5.5 years, children’s performance IQ was assessed using the WPPSI-III (Wechsler Preschool and Primary Scale of Intelligence 3rd Edition).

### Statistical analysis

Missing data were treated using full information maximum likelihood estimation with robust standard errors [50]. Excluding individuals with missing data from our analyses did not significantly alter our results. We first used confirmatory factor analysis (CFA) to identify the latent structure underlying children’s language skills at 3 and 5.5 years.

Next, we performed cross-lagged structural equation models (SEM) [51] to simultaneously examine complex relationships between latent variables (language skills at age 3 and 5.5 years) and manifest variables (i.e., SDQ IH symptoms scores) at each time point [51, 52]. Particular attention was paid to the longitudinal cross-lagged associations between different language skills and IH symptoms (Table 2). We also examined longitudinal cross-lagged associations between language skills at 3 and 5.5 years and between SDQ IH symptoms scores at 3 and 5.5 years (i.e., stability paths), as well as concurrent associations (i.e., the correlation between variables measured at the same time) (Additional file 1: Table S1).

**Table 2:** Standardized parameter estimates of the cross-lagged associations and fit indices of the four structural equation models (EDEN study; *N* = 1459)

		Model 1^a^			Model 2^b^			Model 3^c^			Model 4^d^		
		Estimate	S.E.	*p*-value	Estimate	S.E.	*p*-value	Estimate	S.E.	*p*-value	Estimate	S.E.	*p*-value
Cross-lagged associations													
Latent variable language at 5.5 years	Hyperactivity/inattention symptoms score at 3 years	0.01	0.02	0.729	0.02	0.03	0.491	0.04	0.03	0.186	0.04	0.03	0.105
Hyperactivity/inattention symptoms score at 5.5 years	Latent variable language at 3 years	**-0.17**	**0.03**	**<0.001**	**-0.17**	**0.03**	**<0.001**	**-0.13**	**0.03**	**<0.001**	**-0.12**	**0.03**	**<0.001**
Model Fit Summary		CFI = 0.992			CFI = 0.993			CFI = 0.992			CFI = 0.992		
		TLI = 0.984			TLI = 0.988			TLI = 0.987			TLI = 0.987		
		RMSEA = 0.025			RMSEA = 0.016			RMSEA = 0.017			RMSEA = 0.017		
		(90 % IC) = (0.017–0.032)			(90 % IC) = (0.009–0.021)			(90 % IC) = (0.011–0.023)			(90 % IC) = (0.011–0.021)		

*P*-values in bold are statistically significant (*p* < 0.01)

^a^Adjusted for recruitment center and age of the child at the time of testing at 3 and 5.5 years

^b^Adjusted for other SDQ scores at 3 years, recruitment center and age of the child at the time of testing at 3 and 5.5 years

^c^Adjusted for performance IQ (WPPSI-III), other SDQ scores at 3 years, recruitment center and age of the child at the time of testing at 3 and 5.5 years

^d^Adjusted for pre- and postnatal environmental factors, performance IQ (WPPSI-III), other SDQ scores at 3 years, recruitment center and age of the child at the time of testing at 3 and 5.5 years

We examined measures of goodness-of-fit, including the comparative fit index (CFI), the Tucker–Lewis index (TLI), the root mean squared error of approximation (RMSEA) and the chi-square test of model fit. CFI and TLI values greater than 0.95 and values of RMSEA less than 0.06 are commonly used to indicate good model fit and were used as cut-offs [53].

In order to test bidirectional relationships between children’s language skills and IH symptoms, we performed four cross-lagged structural equation models by gradually adding predictors of cognitive development. In Model 1, we examined the relationship between language skills and SDQ IH symptoms scores without correcting for confounding variables. In Model 2, we included the other SDQ scores at 3 and 5.5 years to take into account the complex relationships between behavioral, emotional and interpersonal difficulties. In Model 3, we also included performance IQ, because the relationship between language skills and SDQ IH symptoms scores could be due to general intelligence rather than specific language difficulties. Finally, in Model 4, we also included pre- and postnatal factors that are relevant to both language skills and behavior problems; i.e., sex, gestational age at birth, birth weight, maternal age at the time of the child’s birth, maternal tobacco and alcohol use during pregnancy, breastfeeding duration, maternal depression during pregnancy, maternal depression after birth, family history of speech and language delay, parental education and income, number of older siblings, cognitive stimulation at age 3, family stimulation at age 5.5 years (*Hypothesis 3*) (Additional file 1: Table S1, Figure S2).

As most language measures were significantly associated with the child’s age at the time of testing as well as the recruitment center, language measures were adjusted for these characteristics in all models. In Models 2, 3 and 4, SDQ scores were allowed to have correlated residuals. We used standardized data because they are less affected by the scales of measurement and can be used to evaluate the relative impact of each predictor [54].

To determine whether a particular aspect of language skills at 3 years was associated with the development of IH symptoms beyond the association attributable to the latent language variable at 3 years, modification indices (i.e., chi-square tests with 1° of freedom) were examined in Model 4 to test whether any residuals of language tests at 3 years were associated with IH symptoms at 5.5 years (i.e., direct effects). We also calculated the total effect of each of the five language tests at 3 years (i.e., the sum of direct and indirect effects *via* the latent language variable at 3 years) on IH symptoms at 5.5 years.

In order to address *Hypothesis 2*, we tested the potential mediation effects of interpersonal difficulties and prosocial behavior by estimating the path from language skills at 3 years to peer relationship problems at 5.5 years, controlling for peer relationship problems at 3 years (Path a), and the path from peer relationship problems at 3 years to IH symptoms at 5.5 years, controlling for IH symptoms at 3 years (Path b), following the recommendations of Cole and Maxwell [55]. The product of Path a and Path b provides an estimate of the mediation by peer relationship problems of the effect of language skills on IH symptoms. The significance of this mediation effect was tested in Model 4 [55, 56]. We tested the mediation effect of prosocial behavior in the same way.

Because our approach was both semi-confirmatory and semi-exploratory, and in order to limit type I error inflation, statistical significance was evaluated using a two-sided design with alpha set a priori at 0.01. All analyses were conducted in Mplus Version 7.1 [57] using the Mplus defaults of delta parameterization and the Maximum Likelihood estimator.

#### Sensitivity analyses

To test the robustness of our findings, we performed several sensitivity analyses using logistic regression models with SDQ scores at 5.5 years (dichotomized at the 85th percentile) as dependent variables (Models A to E; Table 3) and language skills at 3 years, pre- and postnatal environmental factors, performance IQ (WPPSI-III), SDQ scores at 3 years, recruitment center and age at the time of testing, as independent variables. Another logistic regression model (Model F; Additional file 1: Table S2) was conducted with the dichotomized language score at 5.5 years (cut-off at < - 1 SD) as the dependent variable.

**Table 3:** Logistic regression models, using dichotomized SDQ scores at 5.5 years (dichotomized at > 85th percentile) as the dependent variables and language skills at 3 years as independent variables

		Language score at 3 years^a^	Models^b^	
		*N* = 775 (84.8 %)	Standardized Estimate	
		Mean (SD)	Estimate	*p*-value
Model A	Emotional symptoms score at 5.5 years ≤ 9 / *N* = 1037 (87.6 %) [ref]	0.01 (0.99)	0.03	0.538
	Emotional symptoms score at 5.5 years > 9 / *N* = 147 (12.4 %)	0.04 (1.05)		
Model B	Conduct problems score at 5.5 years ≤ 7 / *N* = 1001 (84.5 %) [ref]	0.05 (0.99)	0.07	0.265
	Conduct problems score at 5.5 years > 7 / *N* = 184 (15.5 %)	-0.13 (1.01)		
Model C	Hyperactivity-inattention score at 5.5 years ≤ 6 / *N* = 992 (83.8 %) [ref]	0.11 (0.94)	**-0.12**	**0.021**
	Hyperactivity-inattention score at 5.5 years > 6 / *N* = 192 (16.2 %)	-0.44 (1.15)		
Model D	Peer relationship problems score at 5.5 years ≤ 3 / *N* = 1016 (85.7 %) [ref]	0.06 (0.97)	0.02	0.806
	Peer relationship problems score at 5.5 years > 3 / *N* = 169 (14.3 %)	-0.20 (1.14)		
Model E	Prosocial behavior score at 5.5 years > 11 / *N* = 997 (84.2 %) [ref]	0.05 (0.97)	0.00	0.937
	Prosocial behavior score at 5.5 years ≤ 11 / *N* = 187 (15.8 %)	-0.11 (1.10)		
		Semantic fluency (ELOLA)		
Model C1	Hyperactivity-inattention score at 5.5 years ≤ 6 / *N* = 992 (83.8 %) [ref]	7.0 (3.8)	0.0	0.991
	Hyperactivity-inattention score at 5.5 years > 6 / *N* = 192 (16.2 %)	6.1 (4.0)		
		Word and nonword repetition (ELOLA)		
Model C2	Hyperactivity-inattention score at 5.5 years ≤ 6 / *N* = 992 (83.8 %) [ref]	7.8 (3.2)	-0.07	0.214
	Hyperactivity-inattention score at 5.5 years > 6 / *N* = 192 (16.2 %)	6.8 (3.5)		
		Sentence repetition (NEPSY)		
Model C3	Hyperactivity-inattention score at 5.5 years ≤ 6 / *N* = 992 (83.8 %) [ref]	7.4 (3.3)	-0.09	0.109
	Hyperactivity-inattention score at 5.5 years > 6 / *N* = 192 (16.2 %)	5.8 (3.2)		
		Picture naming (ELOLA)		
Model C4	Hyperactivity-inattention score at 5.5 years ≤ 6 / *N* = 992 (83.8 %) [ref]	7.1 (1.8)	-0.06	0.220
	Hyperactivity-inattention score at 5.5 years > 6 / *N* = 192 (16.2 %)	6.4 (2.3)		
		Comprehension of instructions (NEPSY)		
Model C5	Hyperactivity-inattention score at 5.5 years ≤ 6 / *N* = 992 (83.8 %) [ref]	8.8 (2.9)	**-0.11**	**0.030**
	Hyperactivity-inattention score at 5.5 years > 6 / *N* = 192 (16.2 %)	7.4 (3.1)		

*P*-values in bold are statistically significant (*p* < 0.05)

^a^Language score (z-score) at 3 years was calculated as the linear combination of the weighted language measures at 3 years (weighted by the loading of each variable on the language factor)

^b^Adjusted for pre- and postnatal environmental factors, performance IQ (WPPSI-III), SDQ scores at 3 years, recruitment center and age of the child at the time of testing at 3 years

## Results

### Structure of language skills

The Confirmatory Factor Analysis model including 2 single latent factors representing language skills measured respectively by the five measures of language skills at 3 years and the five measures of language skills at 5.5 years provided an excellent fit to the data: CFI = 0.992, TLI = 0.988 and RMSEA = 0.031 (95 % CI [0.021, 0.041]). Both latent variables provide a general index of language skills, encompassing phonology, syntax, lexicon and conceptual knowledge, using both receptive and expressive modalities.

The stability of language skills between 3 and 5.5 years in our models was found to be high (β = 0.76). This result supports the idea that the latent variables for language at 3 years and 5.5 years reflect similar constructs.

### Structural equation model

Our structural equation models displayed excellent fit to the data (see fit indices in Table 2).

In the four consecutive SEMs, IH symptoms at 5.5 years were significantly predicted by language skills at 3 years (standardized estimate in Model 4: β =−0.12, SE = 0.04, *p*-value = 0.002). Language skills at 5.5 years were not associated with SDQ IH symptoms scores at 3 years (standardized estimate in Model 4: β = 0.04, SE = 0.03, *p*-value = 0.105) (Table 2 and Additional file 1: Figure S2).

Next, we tested whether the association between language skills at 3 years and IH symptoms at 5.5 years was mediated by peer relationship problems or prosocial behavior (*Hypothesis 2*). We found no evidence of such mediation (peer relationship problems: Wald test = 0.48; *p*-value = 0.490; prosocial behavior: Wald test = 0.24; *p*-value = 0.621).

Beyond the effect of the latent variable representing language skills at 3 years on IH symptoms at 5.5 years, there were no significant direct effect of language tests at 3 years on IH symptoms at 5.5 years. The ranks of the total effects were the following: comprehension of instructions (β =−0.13), sentence repetition (β =−0.11), picture naming (β =−0.08), word and nonword repetition (β =−0.04) and semantic fluency (β =−0.03).

### Sensitivity analyses

Examining language skills and IH symptoms when they were dichotomized to reflect potentially clinically significant problems (language skills: < -1 SD; SDQ IH symptoms scores > 6; i.e., 85th percentile) did not alter the significance of our results. Children with IH symptoms at 5.5 years which may reflect clinically significant problems (SDQ IH symptoms score above 6; 16.2 % of our sample) had significantly lower scores on language skills at 3 years (Model C: standardized estimate = -0.12; *p*-value = 0.021; Table 3). One language test at 3 years (comprehension of instructions) was significantly associated with clinically significant IH symptoms at 5.5 (Models C5; Table 3; β = -0.11, *p*-value = 0.030). The dichotomized (at < - 1 SD) language score at 5.5 years was not associated with SDQ IH symptoms scores at 3 years (Model F; standardized estimate = 0.03; *p*-value = 0.601; Additional file 1: Table S2).

As individual differences in language skills (β = 0.77) were found to be more stable than individual differences in IH symptoms (β = 0.47), we conducted a sensitivity analysis a) removing the effect of language skills at 3 years on language skills at 5.5 years and b) removing the effect of IH symptoms at 3 years on IH symptoms at 5.5 years in Model 4. Under these conditions, the effect of language skills at 3 years on IH symptoms at 5.5 years (β = -0.18, SE = 0.08, *p*-value < 0.001) was much greater than the effect of IH symptoms at 3 years on language skills at 5.5 years (β = -0.01, SE = 0.02, *p*-value = 0.792) (Wald test of the difference = 7.61; *p*-value = 0.006), implying that the effect of language skills at 3 years on IH symptoms at 5.5 years is unlikely to be explained by differences in cross-time stability.

After verifying that the measurement parameters of the latent variables were sex-invariant, we used a multiple-group structural equation model stratified by sex and found no significant sex differences (Wald test = 0.32; *p*-value = 0.575) in the effects of language skills at 3 years on IH symptoms at 5.5 years (males: β = -0.09, SE = 0.06, *p*-value = 0.115; females: β = -0.14, SE = 0.06, *p*-value = 0.020). Yet, males had significantly higher SDQ IH symptoms scores at 3 and 5.5 years (β_males_ - β_females_ = 0.21, *p*-value < 0.001; and 0.29, *p*-value < 0.001; respectively) and significantly lower language skills at 3 years but not at 5.5 years (β_males_ - β_females_ = -0.33, *p*-value < 0.001; and -0.10, *p*-value = 0.135; respectively).

## Discussion

Prior studies indicate high levels of comorbidity between ADHD and language impairment [7, 16, 17], highlighting the importance of longitudinal research in testing different hypotheses on the nature of these associations. Based on a large (*N* = 1459), prospective, mother-child cohort, our study confirms the asymmetrical relationship between language skills and IH symptoms during the preschool period. Early language skills not only predict later language skills but also later IH symptoms. Our results are consistent with prior findings [13, 14].

Regarding the specific nature of the influence of early language on later IH symptoms, we found that the comprehension of instructions tests at 3 years was most strongly related to IH symptoms at 5.5 years (Models 4: β = -0.13 and C5 : β = -0.11, *p*-value = 0.030). Moreover, a nonsignificant trend was observed between the sentence repetition test at 3 years and IH symptoms at 5.5 years (Model C3: β = -0.09, *p*-value = 0.109 and Model 4: β = -0.11). Interestingly, the other three language tests at 3 years, which only involved single words (semantic fluency, word and nonword repetition, and picture naming), were not associated with IH symptoms at 5.5 years. Thus, these results suggest that early syntactic ability is the language domain most strongly associated to the development of IH symptoms. At first sight, these results seem to differ from those reported by [18], which mentioned expressive language deficits as the main precursor of inattention symptoms, in children with a history of speech-language impairment. Yet, results reported in their Figure 1 suggest that receptive language was also affected in these children. The comparison between the two studies is hindered by the fact that Snowling et al. [18] have chosen to group language variables into receptive and expressive components, rather than into word-level *versus* sentence-level, or than reporting the results of each test. Thus our results may be more similar than their reporting suggests. Overall, our finding that sentence- rather than word-level language skills predict IH is consistent with the view that language-based self-regulation mediates this relationship (*Hypothesis 1*), if one takes the plausible view that language-based regulation requires formulating propositional phrases, as opposed to just single words.

Among the other hypotheses that have emerged to explain the directional relationship between early language skills and later ADHD symptoms, our results do not support the interpersonal difficulties hypothesis (*Hypothesis 2*) as the main explanation. Indeed, the link between language skills at 3 years and IH symptoms at 5.5 years was not found to be mediated by interpersonal difficulties. This result differs from the study conducted by Menting et al. [25], but is not necessarily contradictory, since a) their study was conducted between ages 6 and 10 whereas our study was conducted during the preschool period and b) their study specifically examined mediation by peer rejection whereas our study examined mediation by broader aspects of interpersonal difficulties (including peer rejection).

Of course, other mediators could be considered, such as working memory which is known to be poorer in ADHD [58–60]. Unfortunately, this study did not include any measure of executive function. The language tests that were used, although they sometimes involved short-term memory (Word and nonword repetition (ELOLA), Sentence repetition (NEPSY) and to a lesser extent Comprehension of instructions (NEPSY)), were relatively light on working memory. Therefore, it is not possible in our study to further investigate the mediating role of working memory, and future studies would benefit in examining it.

Our results do not either support the idea that asymmetrical relationships might reflect the effect of environmental factors becoming manifest in different domains at different ages (*Hypothesis 3)*. We found a small decrease (14 %) in the estimates of the effect of language skills on later IH symptoms when comparing unadjusted models to models that were adjusted for a broad range of pre- and postnatal factors. Thus, the asymmetrical relationship is essentially unaffected by the effects of a broad range of environmental factors.

Overall, our results suggest that language difficulties in the syntactic domain precede the development of IH symptoms during the preschool period. One explanation would be that language difficulties represent an early marker of ADHD, i.e., an early expression of the disorder [14, 15]. Another explanation would be that early language difficulties in the syntactic domain may play a causal role in the development of IH symptoms. Indeed, in line with prior findings [10], children with ADHD may experience unexpected difficulties comprehending more complex information than children without ADHD. They may also have difficulties formulating sophisticated self-directed instructions to regulate their own behavior.

### Strengths and limitations

Strengths of our study are the longitudinal design, the large sample size and the usage of validated language tests and questionnaires.

One possible limitation of our analysis is that IH symptoms were assessed using behavior rating scales completed by parents (SDQ), and could reflect reporting bias. More than one source of information and particularly preschool teacher’s ratings of IH symptoms would have also been useful, as the child’s ability to attend and concentrate and remain at his/her desk or place in the circle is usually more fully tested in the preschool setting [61]. In addition it is also possible that the parent may be rating the child’s difficulty in following instructions and verbally stated demands – that is problems understanding or retaining what the parent is asking or demanding - as a symptom of inattention and hyperactivity. Further studies will have to confirm our findings by measuring IH symptoms through teachers’ or other non-parental raters. Second, some SDQ scores (SDQ emotional symptoms and peer relationship problems at 3 and 5.5 years; SDQ peer relationship problems at 3 years) and language tests (semantic fluency and picture naming at 3 years) had relatively low internal consistency (<0.70), as is often the case with scales assessing complex constructs based on a limited number of items (*e.g*., 5 for the SDQ scores). Third, our study was not suited to determine whether verbal self-regulation mediates the effects of language skills on later IH symptoms because no direct measurement of self-regulation skills was available in our study. Fourth, developmental trajectories of children’s language and IH symptoms are complex and intertwined. Further studies focusing on individual trajectories of language and behavioral development are warranted. Finally, the rate of maternal depression in this sample was relatively high. However it must be underlined that the cut-off of the CES-D (i.e., 16) used to define depression at 3 and 5 years was chosen to increase sensitivity to detect mothers at high risk of having clinical depression (including subthreshold forms) [62], which may have an impact on maternal reporting of child behavior problems [63].

## Conclusions

During the preschool period, poor language skills seem to be associated with later IH symptoms. Our results suggest that language difficulties in the syntactic domain precede and may favor the development of IH symptoms during the preschool period. Detection of language delay in early communication therefore warrants follow-up of the child’s self-regulation development. However, to date, no study has specifically examined whether the identification and early treatment of language difficulties during the school years could reduce later emergence of IH symptoms (except an incidental finding in Glogowska et al. [64]). Further studies are needed to establish whether this relationship is really causal, and to determine whether the identification and early treatment of language difficulties during the preschool period might help reduce the later emergence of IH symptoms.

## Additional files

Additional file 1: Table S1. Standardized parameter estimates of the structural models (Model 4; *N* = 1459). **Table S2.** Logistic regression model (Model F), using dichotomized language scores at 5.5 years (dichotomized at < - 1SD) as the dependent variables and SDQ scores at 3 years as independent variables. **Figure S1.** Flowchart. **Figure S2.** Cross-lagged associations between language skills and the SDQ scores between the ages of 3 and 5.5 years in the EDEN mother-child cohort (*N* = 1459) [Model 4; see Table 3 for details on the model]. (DOC 373 kb)
Additional file 2: The dataset supporting the conclusion of this article. (XLSX 148 kb)

## Abbreviations

ADHD: Attention Deficit/Hyperactivity Disorder
CFI: Comparative Fit Index
EDEN: Etude des Déterminants pré et postnatals précoces du développement et de la santé de l’ENfant
ELOLA: Evaluation du Langage Oral de L’enfant Aphasique
IH: Inattention/Hyperactivity
IQ: Intelligence Quotient
NEPSY: A Developmental NeuroPSYchological Assessment
RMSEA: Root Mean Squared Error of Approximation
SDQ: Strengths & Difficulties Questionnaire
SEM: Structural Equation Modeling
TLI: Tucker–Lewis Index
WPPSI-III: Wechsler Preschool and Primary Scale of Intelligence 3rd Edition
