# Supplementary material for: Relationship between early language skills and the development of inattention/hyperactivity symptoms during the preschool period: Results of the EDEN mother-child cohort
Source: BMC Psychiatry. 2016 Nov 8;16:380. doi: 10.1186/s12888-016-1091-3 (PMC5100106; doi:10.1186/s12888-016-1091-3)

Additional file 1

Supplementary Table 1. Standardized parameter estimates of the structural models (Model 4; N = 1,459).

|  |  | **Model 4***^1^ | | | | |
| --- | --- | --- | --- | --- | --- | --- |
|  |  | Estimate |  | **S.E.** |  | **p-value** |
| **Cross-lagged associations** |  |  |  |  |  |  |
| Latent variable language at 5.5 years | Emotional symptoms score at 3 years | -0.01 |  | 0.03 |  | 0.685 |
|  | Conduct problems score at 3 years | -0.03 |  | 0.03 |  | 0.212 |
|  | Hyperactivity/inattention symptoms score at 3 years | 0.04 |  | 0.03 |  | 0.105 |
|  | Peer relationship problems score at 3 years | 0.01 |  | 0.03 |  | 0.606 |
|  | Prosocial behavior score at 3 years | -0.03 |  | 0.03 |  | 0.220 |
| Emotional symptoms score at 5.5 years | Latent variable language at 3 years | 0.00 |  | 0.04 |  | 0.929 |
| Conduct problems score at 5.5 years | Latent variable language at 3 years | -0.02 |  | 0.04 |  | 0.591 |
| Hyperactivity/inattention symptoms score at 5.5 years | Latent variable language at 3 years | **-0.12** |  | **0.04** |  | **0.002** |
| Peer relationship problems score at 5.5 years | Latent variable language at 3 years | -0.07 |  | 0.04 |  | 0.123 |
| Prosocial behavior score at 5.5 years | Latent variable language at 3 years | 0.03 |  | 0.04 |  | 0.560 |
| **Stability paths** |  |  |  |  |  |  |
| Latent variable language at 5.5 years | Latent variable language at 3 years | **0.77** |  | **0.03** |  | **<0.001** |
| Emotional symptoms score at 5.5 years | Emotional symptoms score at 3 years | **0.39** |  | **0.03** |  | **<0.001** |
| Conduct problems score at 5.5 years | Conduct problems score at 3 years | **0.49** |  | **0.03** |  | **<0.001** |
| Hyperactivity/inattention symptoms score at 5.5 years | Hyperactivity/inattention symptoms score at 3 years | **0.47** |  | **0.03** |  | **<0.001** |
| Peer relationship problems score at 5.5 years | Peer relationship problems score at 3 years | **0.38** |  | **0.03** |  | **<0.001** |
| Prosocial behavior score at 5.5 years | Prosocial behavior score at 3 years | **0.38** |  | **0.03** |  | **<0.001** |
| **Concurrent associations** |  |  |  |  |  |  |
| Latent variable language at 3 years | Emotional symptoms score at 3 years | 0.07 |  | 0.03 |  | 0.044 |
|  | Conduct problems score at 3 years | -0.06 |  | 0.03 |  | 0.089 |
|  | Hyperactivity/inattention symptoms score at 3 years | **-0.09** |  | **0.03** |  | **0.008** |
|  | Peer relationship problems score at 3 years | **-0.15** |  | **0.04** |  | **<0.001** |
|  | Prosocial behavior score at 3 years | **0.12** |  | **0.04** |  | **0.001** |
| Latent variable language at 5.5 years | Emotional symptoms score at 5.5 years | -0.07 |  | 0.05 |  | 0.112 |
|  | Conduct problems score at 5.5 years | -0.01 |  | 0.05 |  | 0.895 |
|  | Hyperactivity/inattention symptoms score at 5.5 years | -0.06 |  | 0.05 |  | 0.242 |
|  | Peer relationship problems score at 5.5 years | 0.00 |  | 0.04 |  | 0.997 |
|  | Prosocial behavior score at 5.5 years | 0.00 |  | 0.05 |  | 0.961 |
| **Loadings** |  |  |  |  |  |  |
| Latent variable language at 3 years | Semantic fluency (ELOLA) | **0.64** |  | **0.02** |  | **<0.001** |
|  | Word and nonword repetition (ELOLA) | **0.62** |  | **0.03** |  | **<0.001** |
|  | Comprehension of instructions (NEPSY) | **0.68** |  | **0.02** |  | **<0.001** |
|  | Picture naming (ELOLA) | **0.71** |  | **0.02** |  | **<0.001** |
|  | Sentence comprehension (NEPSY) | **0.69** |  | **0.02** |  | **<0.001** |
| Latent variable language at 5.5 years | Non Words Repetition (NEPSY) | **0.56** |  | **0.02** |  | **<0.001** |
|  | Sentence Repetition (NEPSY) | **0.71** |  | **0.02** |  | **<0.001** |
|  | Information (WPPSI-3) | **0.84** |  | **0.02** |  | **<0.001** |
|  | Vocabulary (WPPSI-3) | **0.70** |  | **0.02** |  | **<0.001** |
|  | Word Reasoning (WPPSI-3) | **0.86** |  | **0.01** |  | **<0.001** |
| *^1^ Adjusted for pre- and postnatal environmental factors, performance IQ (WPPSI-III), SDQ scores at 3 years, recruitment center and age of the child at the time of testing at 3 and 5 years. | | | | | | |
| P-values in bold are statistically significant (p < 0.01). | | | | | | |

Supplementary Table 2. Logistic regression model (Model F), using dichotomized language scores at 5.5 years (dichotomized at < - 1SD) as the dependent variables and SDQ scores at 3 years as independent variables.

|  |  | Language score at 5.5 years ≥ - 1 SD [ref] |  | Language score at 5.5 years < - 1 SD |  | **Model F***^1^ | | |
| --- | --- | --- | --- | --- | --- | --- | --- | --- |
|  |  | N = 958 (84.8%) |  | N = 171 (15.2%) |  | Standardized Estimate | | |
|  |  | Mean (SD) |  | Mean (SD) |  | Estimate |  | p-value |
| Emotional symptoms score at 3 years |  | 6.80 (1.61) |  | 6.77 (1.59) |  | -0.05 |  | 0.281 |
| Conduct problems score at 3 years |  | 6.04 (1.99) |  | 6.92 (2.14) |  | 0.10 |  | 0.052 |
| Hyperactivity/inattention symptoms score at 3 years |  | 4.31 (2.24) |  | 5.44 (2.28) |  | 0.03 |  | 0.619 |
| Peer relationship problems score at 3 years |  | 2.37 (1.42) |  | 2.80 (1.53) |  | 0.04 |  | 0.440 |
| Prosocial behavior score at 3 years |  | 12.78 (1.64) |  | 12.28 (1.65) |  | 0.03 |  | 0.548 |
| *1 Language score (z-score) at 3 and 5.5 years were calculated as the linear combination of the weighted language measures at 3 years and 5.5 years respectively (weighted by the loading of each variable on the language factor at 3 and 5.5 years). | | | | | | | | |
| *2 Adjusted for pre- and postnatal environmental factors, performance IQ (WPPSI-III), language score at 3 years, recruitment center and age of the child at the time of testing at 3 and 5.5 years. | | | | | | | | |

Supplementary Figure 1. Flowchart.


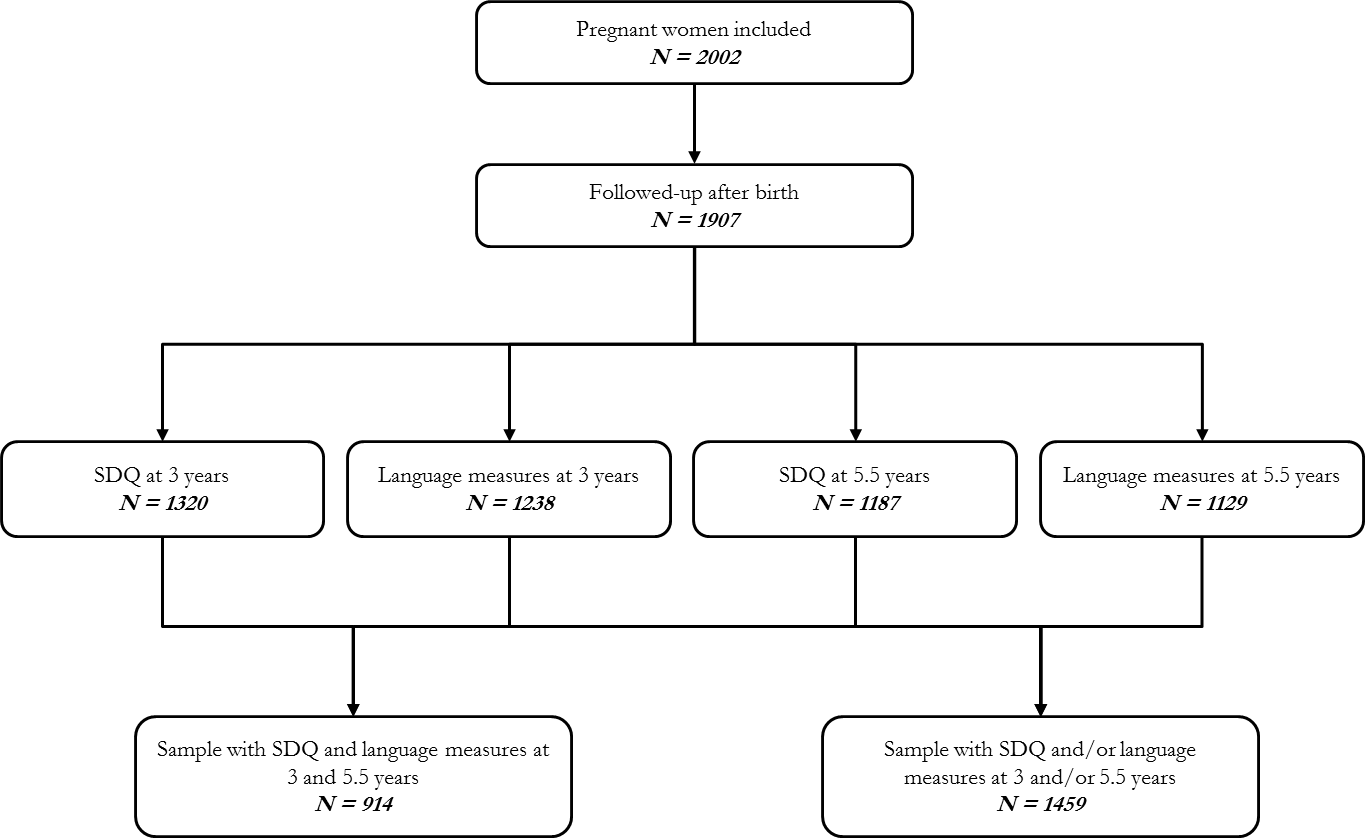


Supplementary Figure 2. Cross-lagged associations between language skills and the SDQ scores between the ages of 3 and 5.5 years in the EDEN mother-child cohort (N=1459) [Model 4; see Table 3 for details on the model].


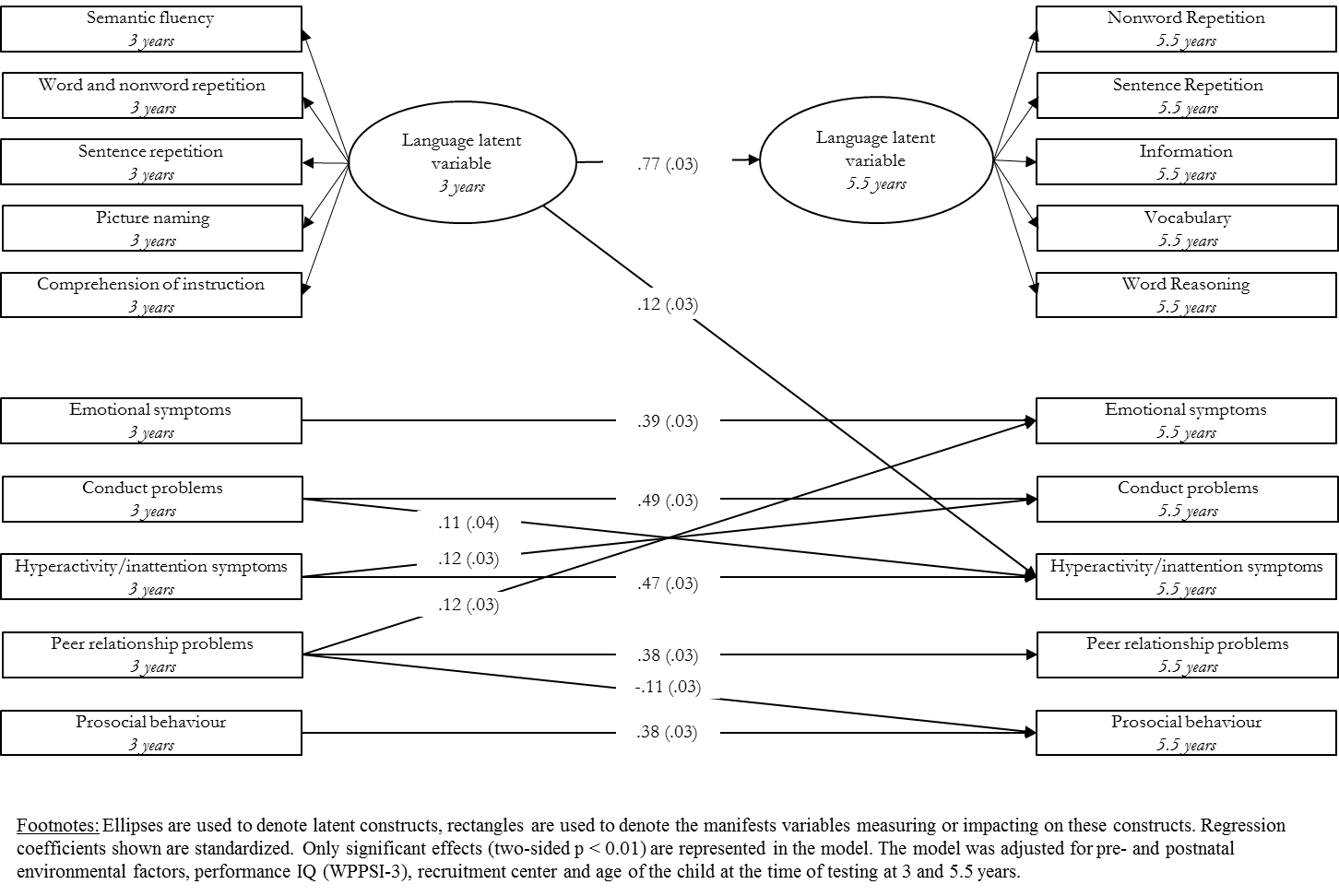

Supplement: Additional file 1: Table S1. — Standardized parameter estimates of the structural models (Model 4; N = 1459). Table S2. Logistic regression model (Model F), using dichotomized language scores at 5.5 years (dichotomized at < - 1SD) as the dependent variables and SDQ scores at 3 years as independent variables. Figure S1. Flowchart. Figure S2. Cross-lagged associations between language skills and the SDQ scores between the ages of 3 and 5.5 years in the EDEN mother-child cohort (N = 1459) [Model 4; see Table 3 for details on the model]. (DOC 373 kb) [file 12888_2016_1091_MOESM1_ESM.doc]
